# Psychosocial implications of tubal ligation in a rural health district: A phenomenological study

**DOI:** 10.1186/1742-4755-8-38

**Published:** 2011-12-16

**Authors:** Prosper M Lutala, Jannie F Hugo, Levi N Luhiriri

**Affiliations:** 1 Department of Family Médecine, Université de Goma, Goma, 2 Avenue Himbi, Congo P.O. Box 204 Goma, Congo; 2 Department of Family Medicine, University of Pretoria, PO Box 53, De Wildt 0251, South Africa; 3 Département de chirurgie, Université Evangélique d'Afrique, Quartier Panzi, Zone Ibanda BP 3323 Bukavu, Congo

**Keywords:** psychosocial implications, tubal sterilization, Congo, tubal sterilization, rural district, mini laparotomy, contraception

## Abstract

**Background:**

Tubal ligation is the most popular family planning method worldwide. While its benefits, such as effectiveness in protecting against pregnancies, minimal need for long-term follow-up and low side-effects profile are well documented, it has many reported complications. However, to date, these complications have not been described by residents in Congo. Therefore, the study aimed at exploring the experience of women who had undergone tubal ligation, focusing on perceptions of physical, psychological and contextual experiences of participants.

**Methods:**

This qualitative study used a semi-structured questionnaire in a phenomenological paradigm to collect data. Fifteen participants were purposefully selected among sterilized women who had a ligation procedure performed, were aged between 30 and 40 years, and were living within the catchment area of the district hospital. Data were collected by two registered nurses, tape-recorded, and transcribed verbatim. Reading and re-reading cut and paste techniques, and integration were used to establish codes, categories, themes, and description.

**Results:**

Diverse and sometimes opposite changes in somatic symptoms, psychological symptoms, productivity, ecological relationships, doctor-client relationships, ethical issues, and change of life style were the major problem domains.

**Conclusions:**

Clients reported conflicting experiences in several areas of their lives after tubal sterilization. Management, including awareness of the particular features of the client, is needed to decrease the likelihood of psychosocial morbidity and/or to select clients in need of sterilization.

## Background

Tubal sterilization is the most practiced method of contraception globally [1-3]. It has been estimated that the procedure has been performed on 190 million women worldwide [4]. Among other advantages, tubal sterilization is a balanced contraceptive method [1]. It offers effective protection against pregnancy, eliminates the need for long-term contraceptive supplies, and has a low risk of exposing women to complications when carried out according to standards. Surgical sterilization is a safe, convenient, easy, and highly effective birth control method for the long term [4]. Furthermore, female sterilization is the best option for countries in the Sub-Saharan region as it can be performed during hospitalization for either normal delivery or caesarean section, saving a return visit and allowing a single recovery period for the surgical procedure and the delivery [5]. Although doing so is not in compliance with the required 40 days postpartum for the procedure, this helps to prevent losing touch with these women in the community, which would then increase the likelihood of an additional high-risk pregnancy that could lead to complications. In Congo, this contraception method seems most appropriate due to the difficult structural, social, and economic conditions prevailing in the health system.

Demographically, Congo is populated by 71,712,867 inhabitants [6]. The total fertility rate is 5.24 children born per woman, and the maternal mortality rate is among the highest globally, with the 2009 estimates reaching 670 maternal deaths per 100,000 live births [6]. The infant mortality rate stands at 129 infant deaths per 1000 live births [6]. In addition, the natality rate is around 48%, with 25.8% young mothers aged 15 to 20 in South Kivu province, where this study was carried out [7].

During the period of armed conflict between 1994 and 2003, the rates of contraceptive use declined in Congo from 15% in 1995 to 5.8% in 2007 [8,9]. The decline had several causes, including the destruction of the health infrastructure and a shortage in family planning products and trained staff [10]. War could also play an indirect role in decisions on family size.

This combination of high birth rates, early first pregnancies, and high maternal and infant mortality rates can be improved by a good family planning program. Unfortunately, the program itself seems underused, with only 7% in Kinshasa, for example, using a contraceptive method [11]. Furthermore, among those using modern methods, the most commonly used are condoms, followed successively by pills, injectables, intrauterine devices (IUD), spermicidal foam, and the diaphragm [11].

Despite some adverse effects reported after tubal sterilization in previous studies [12,13], the advantages of sterilization stated above far outweigh the problems caused by the procedures in women.

This study was conducted in Kaziba, a rural, remote area of the South Kivu province. Kaziba is a very poor area in which the main businesses rely on trades. It has an area of 195 square kilometers and a population of 28,223 inhabitants [14]. The area is served by 3 medical doctors and 105 nurses of different levels among a total of 278 health staffs. Malaria, anemia, and malnutrition are significant issues in the area. In addition to the small-scale farming of different crops (bananas, sorghum, maize, beans, potatoes, coffee, quinidine), and animal breeder, the artisanal production of gold also occurs in the area [14]. Kaziba is dominated by the Protestant (Pentecostal) denomination due to a history of missionaries working in the Eastern Congo since 1919. Roman Catholicism is the next largest religious group, with estimates reaching 25% of the population.

Sterilization procedures on healthy women are expected to have a side-effects free profile and provide the benefit of increased quality of life. However, several studies have reported menstrual, somatic, affective, relational, or psychological problems following sterilization in women [4,15]. To our knowledge, no study has been conducted in Kaziba. Furthermore, a high proportion of previous studies [4,12] used quantitative designs and could not deepen the understanding of the victims per se. The mechanisms to explain those complaints used different variables from study to study, but a common point lay in the high morbidity following the procedure.

So far, few existing reports have focused on psychosocial aspects of this morbidity, and none recently in MEDLINE (Medical Literature Analysis and Retrieval System Online) used a qualitative approach to explore the experiences of clients after sterilization. Therefore, the study aimed at exploring the experience of women who had undergone tubal ligation, focusing on perceptions of physical, psychological and contextual experiences of participants.

## Methods

A qualitative study was completed using a phenomenological approach [16] through individual interviews. This approach helps us to understand social phenomena in natural settings (in participants' own territory, own language, and on their own terms); giving emphasis to the meaning, experience, and view of all the participants [17].

The phenomenological approach is a doctrine introduced by Edmond Husserl that has a starting point of asking, "How can truth be apprehended subjectively?" [16]

Fifteen women were purposefully selected from all available sterilized women with regard to their age ranges, post-operative experience period, and habitat. The inclusion criteria were age between 30 and 40 years, 3 to 7 years post-tubal-sterilization experience, living within the hospital catchment area, and willingness to participate in the study. Before age 30, tubal ligation was rare in our setting and after 40 years old pre-menopausal symptoms could be confused with some complications related to sterilization. The time of the experience was also chosen to avoid early complaints after the procedure, which could be due to the procedure or anesthesia, but early enough to decrease a recall bias when reporting the experience. There was also a need to deepen our understanding by having the opportunity to share with participants whenever necessary within the surroundings of the facilities. Kaziba was selected due to high demand for tubal sterilization by couples in the region. Women who had been unwillingly ligated because of few medical conditions like eclampsia or madness were excluded from the study.

Data were collected using individual interviews by two trained registered nurses conversant in Mashi, Kiswahili, and French, the three commonly spoken languages in the region. The interviews were conducted either in the home of participants during their free time or at the hospital after closure of out-patient department. Our exploratory question was "*You were operated on for tubal sterilization. What are your impressions of your life after the operation*?'" From there, probing questions were used to explore other dimensions related to tubal sterilization and elucidate ideas from the participants' perspective. The interviews were continued until reaching a saturation of ideas. At the end of the interviews, socio demographic data were collected. For seven participants, a second interview was scheduled to get clarification on some "unspoken" thoughts or to probe some unclear ideas. The interviews averaged 90 minutes in duration. They were audio taped and transcribed verbatim by the second research assistant.

We listened to each tape at least three times to gain a good understanding of the content, to construct codes, and to group the women's statements into categories. Links between categories with special attention on similarities, differences, opposition or contrasts, and comparison between written data and recorded data gave us the means to detect themes. Practically, Tesch's [18] steps were used to link ideas throughout the data; that is, to gain an understanding of the whole interview by reading the entire summary carefully. Ideas were written down as they came to mind. We focused on one interview that seemed to stand out as being clearer, more concise, and more interesting. From there, we asked ourselves "What is it all about here?" or "What is the underlying meaning?" The decided-upon meaning was recorded and the same procedure was continued for all 15 interviews. Similar ideas were grouped and arranged as strong, medium, and weak expressions of ideas depending on how they were related to the psychosocial implications of tubal sterilization. Every code in the text was given a number and recorded in the whole text. The most expressive ideas were defined as categories. We drew a line between categories, and relationships between them were established until we reached themes.

On the other hand, content analysis through cut and paste and integration of themes as described by Berelson [19] was used to integrate data by the principal investigator. The results of these two analytical approaches were compared to observe similarities. Codes, categories, and themes were written in two copies and handed to two data collectors/research assistants for validation. The two approaches were cross-validated to find similarities and differences and were discussed among the three researchers. Their comments and suggestions were integrated after discussion with the principal investigator to enrich the data. In a second procedure, seven participants were purposefully selected based on their significant experiences with tubal ligation were consulted with a draft of the themes, categories, and codes and asked for their feedback. For validation, a doctoral-level anthropologist with long experience in qualitative methods was also given the raw materials and the results and asked to comment on the draft and on the way in which the research was conducted.

Participants were asked to voluntarily participate after being given a full explanation of the pros and cons of the study. An informed consent form was signed before starting the interviews. All collected data were kept securely, and confidentiality was kept throughout the whole process. Ethical clearance was sought and granted by the Research, Ethics, and Publications Committee of the Faculty of Medicine, Medical University of Southern Africa (N0. MP 101/2000). This research is part of a dissertation submitted to the same university by the principal investigator in partial fulfillment of the requirements for the Master of Medicine degree.

## Results

### Socio-demographic data

As shown in Table 1, ages of the 15 participants ranged between 35 and 40 years (median 38), most were married, and most attained secondary school level education. The average number of years married was 17, most were 3 years post-sterilization, all were Protestant, and social levels were almost equally distributed between I and II. Decision making (consent) for the procedure was made either by medical staff for slightly low than half of them, the female participant for third of the participants, and their husbands for the remaining. (Table 1)

**Table 1:** Socio demographic data of respondents

Participants	1	2	3	4	5	6	7	8	9	10	11	12	13	14	15
Age	**39**	**40**	**40**	**40**	**40**	**35**	**38**	**40**	**36**	**38**	**39**	**38**	**38**	**37**	**37**
**Marital status**	M	M	W	M	M	M	M	M	M	M	M	M	M	M	M
Study level	**O**	**S**	**P**	**P**	**P**	**P**	**S**	**S**	**P**	**S**	**S**	**S**	**S**	**S**	**S**
**Years of marriage**	19	19	22	24	25	16	15	25	17	21	20	19	19	20	17
Parity	**9**	**7**	**6**	**4**	**6**	**6**	**6**	**10**	**3**	**6**	**8**	**5**	**7**	**3**	**5**
**Gender ratio (m/f) of living children**	1/3	4/3	3/3	3/1	4/2	5/1	3/3	6/4	4/1	2/2	3/5	4/1	2/5	1/1	3/2
Years after tubal ligation	**3**	**3**	**3**	**5**	**3**	**3**	**3**	**5**	**3**	**4**	**3**	**5**	**5**	**1**	**5**
**Religion**	Pr	Pr	Pr	Pr	Pr	Pr	Pr	Pr	Pr	Pr	Pr	Pr	Pr	Pr	Pr
Socioeconomic level	**I**	**I**	**I**	**I**	**I**	**I**	**I**	**I**	**II**	**II**	**II**	**II**	**II**	**II**	**II**
**Decision-maker for tubal sterilization**	Wi	Wi	Me	Me	Me	Me	Me	Me	Me	H	Wi	Wi	Wi	H	H

M: married, W: widowed, O: illiterate, P: primary school; S: secondary school, P.: protestant, I and II: see socio-economical levels in methodology; W: wife; Me: medical indication, H: husband consent. M: male, f: female

### Themes and categories

Main themes and categories are summarized in Table 2. Those themes are:

**Table 2:** Themes identified

Area	Related to	Themes
**1. Somatic symptoms**	Physical symptoms that increased, disappeared, or worsened.	1. Menstrual disorders 2. Backache 3. Abdominal pains 4. Undifferentiated symptoms 5. Pregnancy-related complaints
**2. Psychological problems**	How the psyche of the client is perceived reacting to sterilization.	6. Guilt-regret 7. Fear 8. Disappointment 9. Peace and satisfaction 10. Change of mind
**3. Productivity**	Ability to work and improve living standard	11. Reduced ability to work 12. Improved social standard 13. Decreased expenses 14. Smooth growth of children 15. Dependency
**4. Human/relational problem**	Relationships of the client in the household, extended family, and society	16. Changed libido 17. Unfaithfulness 18. Family support
**5. Doctor-client relationships**	Relationship of doctor-client in decision making and counseling	19. Lack of informed consent 20. Ineffective counseling 21. Unilateral decision

### Somatic symptoms

Somatic symptoms varied among participants. For some they were increased, whereas they decreased or remained unchanged for others.

The respondents were of the opinion that menstrual problems were very common after tubal sterilization. Those troubles included also pain during menstruation. And one declared:

*...I had a lot of pains in my back during menstruation..*. (Participant 3, widowed, 40 years old, medical decision-making and with 6 children).

Pain was perceived differently by the participants. For some, the burden of pain was outweighed by the advantages resulting from tubal sterilization explained by absence of long inactive period.

.... *in spite of these pains, ..... I am free of being bedridden for a long time like I used to go through during pregnancies (Participant 5, married, 40 years old, medical indication, 6 children*.

This is why one considered tubal ligation a kind of 'regeneration' or a "new beginning."

*I have a lot of pains during my period, but I am able to live again after tubal sterilization *(participant 3, widowed, 40 years old, medical decision-making and with 6 children).

In addition to painful menstruations, another group is experiencing isolated backaches.

Sterilization is seen to cause backaches in sterilized women; however, the backaches are tolerable for most participants in comparison with the suffering generated by recurrent anemia previously.

*Even with back pains, it is better because with pregnancies I got postpartum anemia five times out of eight deliveries (participant 15, married, 37 years old, 5 children and decision made by husband)*.

Abdominal pains were reported by other participants as an outcome of sterilization.

*... Am suffering from various illnesses, among them abdominal pains! (Participant 1 married, 39 years old, 4 alive children among 9 born, self-decision-making)*.

These somatic symptoms were sometimes very disturbing, undifferentiated, and non-specific even difficult to link to the procedure.

One participant

*................ but now I am more often sick from diverse things or conditions. The sterilization gave me troubles, even swelling of the belly. (Participant 5 married, 40 years old, 6 children, medical indication)*.

The extent and number of somatic problems was so great that they pushed some participants to reconsider the procedure and to suggest re-opening their tubes.

*After my ligation,....., I start having periods two or three times per month, and back pains, so I am thinking that the sterilization has caused all this suffering. Even a swollen abdomen! ... Could go to re-open those tubes(Participant 5 married, 40 years old, medical indication, 6 children)*.

For other participants, sterilization led to a decrease in physical complaints. Changes were so obvious that some compared going through tubal sterilization as "coming back to life" and causing the disappearance of abdominal pains or the decrease of back pain and pain during menstruation.

*...............I have minor back pains and pains during periods, but...... I have come back to life[ after tubal ligation].(*participant 3, widowed, 40 years old, medical decision-making and with 6 children)

Another declared:

*.....As soon as I was conceiving I use to have back pain and lower abdominal pains. Now finally I have some relief*. (Participant 13, married, 38 years old, 7 children, self-decision-making).

A third woman added:

*The positive thing is that the lower belly pain I used to have has completely disappeared*.

Despite recognizing that there were not many changes after sterilization, one participant acknowledged that tubal sterilization totally relieved her pains during menstruation. It also satisfied the woman and her close relatives, who felt she was a burden due to incapacitation during pregnancies.

*I can say that there have been no significant changes, apart from the period's pains, which disappeared, and there are no longer lower belly pains..., the weakness and inability to work after conceiving ..*...*can work know for the children I have already. (*(Participant 13, married, 38 years old, 7 children, self-decision-making).

### Feeling of guilt, regrets and disappointment

Participants' experienced psychological problems in a number of ways after being sterilized, such as the belief that health problems were attributed to the procedure, disrespect to God's plan for reproduction, and disturbance in the relationship with their partner (husband).

One participant (not informed about the pros and cons of tubal ligation) felt guilt about having been sterilized after experiencing uncontrolled diabetes mellitus. She tried to link all her diabetic issues to the procedure.

*I am suffering now after being ligated due to uncontrolled diabetes. ...ligation has worsened my condition *[meaning diabetes]......' *(Participants 5, married, 40 years old, medical indication, 6 children)*

The guilt was so important to one participant (operated in emergency without prior counseling after an) that she could never advise or encourage someone else to undergo sterilization because sterilization did not stop her from getting sick.

*I can never advise someone to get ligated because I have seen no advantage from it. If I had ceased to get ill, I could advise it. But, I have no peace after tubal sterilization*. *(Participant 5 married, 40 years old, medical indication, 6 children)*.

Another participant considered sterilization contrary to God's will when done only for family planning purposes.

*Sterilization is a fault, a sin. If you have difficulties [meaning health problems], then in this case you are ok. We are supposed to give birth normally ... as many as God provides! (Participant 8 married, 40 years old, medical indication, 10 children)*.

Another group experienced marital problems either due to lack of sexual pleasure and/or pains during sexual intercourse which led to erratic sexual intercourse.

*Since the ligation, there is nothing more I can say about the back pains. Most of the time, I don't want to do that thing anymore *[meaning sexual intercourse]. *It doesn't give me any pleasure, especially when I think about this pain. Sometimes I do it just by formality, but it's no longer important for me. (Participant 14, married, 37 years old, decision-maker: husband, 3 children)*

Regrets stemming from the procedure occurred for several reasons, including change in family situation, quarrels within the family, and the desire to have more children after sterilization.

*This led the husband to consider, either getting a second wife of child-bearing age or quarrying in the household*.

*First, my husband refuses to pay for my medical care; second, he is exploring ways to have others' children outside. Currently, myself, I have many illnesses. (Participant 1 married 39 years old, own decision-making)*.

Others doubt and regret the sterilization even though they acknowledged the benefits of the operation. They are uncertain if their family size will remain stable due to the high mortality rate for children in Kaziba, and some even express the extreme position of wanting to reopen their tubes.

*...... But I cannot advise it. With the wars every day in this province, I am not sure of the number I will have once they are adults*.

Participants expressed fear and disappointment for several reasons, such as the inability to have a child and perception of tubal sterilization as sinful when used as a family planning method.

...after sterilization, I had no worries, but now I want another child. (Participant 12, married, 38, own decision-making, no clear knowledge about tubal ligation prior operation)

### Peace and satisfaction

After tubal sterilization, participants reported having either physical or mental relief that gave them a sense of satisfaction due to the absence of pregnancy-related problems.

...the main change I am experiencing is relief .... I feel really relieved. I have no health problem and I am fine. (Participant 11, 39, married, self-decision making)

Others feel relieved of fear of an unplanned pregnancy. One who use to spend months on the bed during the previous pregnancies acknowledged:

*..... Since then, I have peace; I am quiet, because I was afraid to get pregnant. (Participant 2, married, 40 years, 7 children, own-decision making)*.

### Productivity

Sterilization decreased the productivity of participants who became unable to work or feed their families because of pain or general body weakness that prevented them from going to the fields. Sterilization was followed by a decrease in expenses, which led to an improved standard of living for children.

... Now I am unable to cultivate because I cannot bend down due to pulsating pains.(participant 8, married, 40 years old, 5 children, medical indication)

These illnesses have kept this woman in their homes instead of going to the fields for the daily farming work.

*........... Since 1996[year of tubal ligation], I have never worked in the field because of illnesses*. (same participant as above).

### Ecological issues (social relationships)

With respect to the households of the participants, some of the social issues raised included decreased libido, continuous menstruation for some, inability to respond to the family's needs by proportionally producing enough food [*The family looks after me for almost everything]*, the husband changing his mind and planning to have another child, reductions of expenses to few children, and conjugal strife

The sexual contacts becomes rare either due to frequent menstruation or decrease of libido in few ladies

One woman elaborated:

I am making him [husband] suffer because I am bleeding frequently; therefore there is no time for sex.((participant 14, 37 years old, 3 children, husband took decision)

The lack of work as started above led one participant to reduce her own self-esteem as breadwinner of the family:

. ...I no longer work, I am always ill, and I am not producing anything. How can I be useful to these children .... (Participant 7, married, 38 years old, 6 children, medical indication)Another was concerned that his husband is planning to get remarried so that he can get more children. She said:

*.....And now, he is focusing his ideas on marrying a second wife who will come and give him children*.

*Since the operation, I have had no peace! ..*.

Such decision and some more, have led to disputes in some households and one to said

, my husband has turned against me and we are having endless problems [ meaning quarrels] at home.(participant 1, married, parity 9 with 3 alive children, self-decision-making)

On the other hand, some praised the benefits of sterilization, which allowed them to take effective care of their children by covering their basic needs related to food, education, reduce maternity related expenses. They have successively acknowledged:

*The ligation is a good thing to maintain my health and the health of the family I already have. (participant 14, married, 3 children, husband decision-making)*.

*..........they grows normally, they eat and they study without difficulties*.

... Allows us to save, to preserve the health of both mother and children, and allows harmonious growth of the children.(participant 13, 38 years old, married, 7 children, self-decision-making)

### Doctor-client communication and ethical issues

The interviews indicated that the way doctors made their decision to perform a tubal sterilization differed; according to the women, this played a role in the aftermath of the procedure. Some gave their full consent to the operation, and others made the decision after being given full information about the procedure but did not discuss it with their partner. Others complained of being operated on unwillingly, and still others underwent the procedure after the decision was made by their doctor, partners, or extended family.

One woman complained about a lack of information:

*Hum! I was ligated early without wanting it. I was forced by my doctor*.

One woman's sister-in-law, who fully supported the brother's family due to his economic difficulties, decided to request sterilization for the woman after consulting the brother. The sister-in-law was able to obtain permission without discussing it with the client.

*It was my husband and my sister-in-law who got me sterilized because she was helping us with everything. I did not talk to the doctor before the operation...., they decided three of them to cutoff maternity-related expenses to my sister-in-law who was tired to support our entire family 9 my husband and 5 children) with her own.....' (Participant 12, married, 38 years old, husband and sister-in-law decision making)*.

Another was not given time to understand the doctor's counseling.

*My first and only contact with the doctor ... I can't remember it well. He only asked me if I wanted to get ligated during the caesarean. I was still hesitating and he proceeded without telling me*. (Participant 6, 35 years old with 3 years post tubal ligation, medical decision-making)

Sometimes the husband was not included in the decision-making process for tubal sterilization, which led to subsequent problems.

*When I was ligated, it was a shared decision of the doctor and me because of several diseases that were leading to surgical operations, but my husband did not know about it. (Participant 1 married 39 years old, own decision-making)*.

Some didn't know even about the procedure

*I have no idea of any contact with the doctor before the procedure*. (Participant 7, 38 years old, married. Medical decision-making)

### Integration of themes

By comparing and integrating the themes developed in this paper, we can say that some participants saw tubal sterilization as a pain/disease reliever because it alleviated back and belly pains and other diverse complaints, which led to stabilization. In such cases, psychologically, women experienced a feeling of peace, decreased stress, and a sense of being "fine." Sterilization also led to increased income by reducing the inability to work and allowing women to devote their money to a small number of children. Considering these advantages, sterilization symbolized a 'resurrection' event. Increased quality of life was achieved by reducing expenses and the number of children to look after.

Negative outcomes included many symptoms, both somatic and psychological, which undermined the well-being of some participants. As a result of sterilization, some women experienced a lack of financial assistance from their husbands, who began looking to have more children outside of wedlock.

Additionally, some women were not well advised or asked their opinion prior to the ligation, and some wished to have another child by reopening their tubes. The negative implications are summarized as trouble with menstrual cycles, dyspareunia, lifelong diseases leading to dependency, and decreased self-esteem. All of these problems aggravated the social conditions of the women.

## Discussion

This study is the first attempt to explore the experiences of women who underwent sterilization in Congo. As such, it explores the meaning that participants attached to this procedure, which could help in anticipating complications and implementing measures accordingly. In countries like Congo, which has a high maternal mortality rate, studies aiming to improve the quality of life (QOL) of women after any family planning method are welcomed.

If the opinion toward tubal sterilization improves, it will become more accepted and lead to improvements in QOL because it prevents high-risk pregnancies. However, the inductive approach of this study does not allow the results to be generalized for all women. Nonetheless, care has been taken to diversify the samples by including diverse participants in the selection of participants and by providing information on the context in which the study took place.

To capture the meaning women ascribed to their experience and to increase the validity of the study, several strategies were used. More than one interview was completed if necessary, fieldwork was conducted in the preferred language of the participants, selection of participants was done on the basis of the women's experiences after sterilization, and data was validated by requesting feedback from participants and a specialist in the field of social science.

The implications of tubal sterilization ranged from clinical conditions to the personal, contextual, and ethical problems within a woman's household. In each woman's experience, contrasting categories and themes were presented in terms of advantages versus disadvantages, gain versus loss, benefits versus harm, and health versus disease (Table 3). This demonstrates how difficult it is to reconcile different views about sterilization. Indirectly, it emphasizes the need to personalize the care of clients from pre-operative counseling to managing postoperative complaints in order to achieve good outcomes.

**Table 3:** Some contrasts in data

I have no peace	**Vs**.	**After sterilization I had peace, I am quiet**
**I start having pains in my back during menstruation**	Vs.	Pains have completely disappeared after sterilization.
... am having 2-3 periods per month.	**Vs**.	**My periods are becoming more regular**
**I am more often sick**	Vs.	I have come back to life
I am unable to cultivate ... I cannot bend down	**Vs**.	**No more back pains!**
**I am often sick**	Vs.	The main change in body is relief
I am not producing anything	**Vs**.	**At least I can produce what I need to feed all**
**It's hard to find what is needed for children**.	Vs.	They grow ...; they can eat and study without difficulty.
I was immobilized in bed ... after conception	**Vs**.	**I am free of being bedridden for months**.
**I am always ill**.	Vs.	Maintain my health & health of my family
**Vs.: versus**		

To understand the implications of this study, we must place the results in a large framework that includes biological factors, the personal needs of the participant, the family, the client-doctor relationship, and the context in which the woman is living [20].

For example, several times, participants saw themselves as useless or non-productive because they were unable to cope with their partner's sexual needs or they failed to cover the nutritional needs of the household. Those two concerns are related, and they could be major contributing factors, or at least players, in some of the subjective complaints expressed by the participants. The meaning attached to a symptom is expressed in terms of its explanatory model and is known to contribute to the genesis of illnesses [21]. The sociological role that a women is expected to play in our study setting is also a factor that could explain the depressive mood some participants expressed in terms of several "unclear" or "undefined" symptoms. According to tradition in Kaziba and elsewhere in the region, there is a gendered distribution of labor; women are, in essence, the breadwinners. Specifically, men are devoted to physical protection and women deal with the nutritional needs of the family by providing food and keeping the husband in a stable physical and psychological state. Failure to fulfill this role leads to insecurity and decreased self-esteem, which is a source of psychosomatic problems, such as anxiety or depression that undermine the well-being of women.

The implications go beyond the household and include many others, such as the mother-in-law, brothers-in-law, neighbors, fellow Christians, church leadership, etc. Each has expectations of the family and may offer advice on how to deal with potential problems, which can lead to the decision for sterilization. First, some individuals, such as in-laws and elders, see themselves as advisors or decision-makers for others who are under their control. They expect others to justify their decisions and to get advice from them in all matters, including sterilization. Second, the doctor-patient relation with its ethical correlates is very questionable in such scenario and reason of great concern. This individual-collective dimension of care is a serious issue and a source of conflict between the Western and African worldviews. To avoid conflict and accommodate the locals, medical providers operating in Kaziba and similar places must reconcile these two views. It is important to preserve the ethical principles of autonomy and confidentiality (biomedical model of care), but also to avoid social disruption that could lead to conflict for the client. From this perspective, common sense and ethical principles have to be balanced, as it is of foremost importance to reflect on the social needs of family-planning clients as well as the principles governing our working rules. However, these questions remain open to further investigation in our setting. Even if decision-making in sterilization is coming from another person, the client has to be aware of the advantages, disadvantages of tubal ligation (which means informed-decision) and availability of others long-term effective contraception methods like vasectomy, intrauterine device and implant.

Non acceptance of sterilization can come from relatives, neighbors, or within their own household. After the procedure, desires such as wanting to remarry or to have more children, or regrets such as misgivings over being sterilized, tend to undermine QOL.

Both the somatic and psychosocial problems observed in this study confirm results from previous studies [22-24]. For example, regret and menstrual disorders were found to be correlated with tubal sterilization [22,24]. While hypothesizing the genesis in terms of etiopathogenesis a disruption in the blood supply, factors such as non-acceptance of the procedure and the early age of the women undergoing the procedure are still the subject of controversy [24]. Several symptoms are linked to psychosocial issues. Fatigue, regret, undifferentiated complaints are some of them. While regret for example have been largely described in previous studies [4], their risk factors such as tubal ligation before 30 years, marital problems, divorce were not directly present in our study. Our main complaints were the desire of another children mostly expressed by the husband and in case that the decision-making process was coming outside the household or from one of the partners. Increased Sexual desire and libido described in previous studies [4], was not found literally in this study where the lack of libido was one concern. But, here the lack of libido was not isolated per se, but connected here to pains (either vaginal or abdominal or both) which were generated by the procedure.

Finally, the role of the doctor in preparing women for the procedure needs to receive more attention. Some statements about the way doctors conducted counseling prior to the procedure are worrisome on ethical grounds. They contrast with Steward's [25] suggested steps for easily reaching a common ground between a doctor and a client seeking treatment or care. He suggested the following steps: (1) develop a shared understanding of the presented problems, (2) understand and agree on the goal of the management plan, (3) explore the expectations of the health provider and client regarding each other's roles, and (4) undertake mutual decision-making in the management of the problem.

The lack of a remembrance of contact with the doctor, the lack of willingness to undergo the procedure, and the minimal contact with the doctor in preparation for the procedure translates insufficient information sharing and effort in reaching a consensus beforehand. Each of the above patients' complaint could translate, in Blitz's [21] terms, to a lack of respect, empathy, caring, and consideration of patients' concerns. The provider has an obligation by law to inform the person of the consequences and possible complications of the procedure [22]. In our setting, it seems that the consent of the client was overlooked by the practitioners; if the client was not informed about the operative procedure, it is obvious that early or late complications could not be discussed. This is a legal and professional issue that must be addressed. There is an ethical requirement to inform the client about what will be done to her body. At the same time, the social context requires that the partner be also involved in the ligation decision. In our study, the partner's involvement was still considered socially acceptable, as reflected in some quotes.

This study adds to the literature exploring the aftermath of sterilization in women in several ways. The role of environment on the well-being of the client and the combination of all factors surrounding her life in response to a stressful tubal sterilization need special emphasis. Beyond this, we must devise management plans within a larger context and include the household, work, neighbors, extended family, etc. Hence, preparation and follow-up must give more attention to these aspects in order to reach well-balanced and healthy post-operative outcomes.

A few questions remain unresolved. The conflict between the autonomy of the client and the need to involve some of the key players in the sterilization decision-making process needs further investigation. The ecological application of informed consent requires a balance between professionalism and the needs of clients while considering local realities. In addition, in a rural, Christian setting with few educated people where growth rates are high and effective use of family planning methods is low, the steps, types, and extent of communication between clients and providers before, during and after tubal ligation could be further explored.

## Conclusions

This study has demonstrated different views in the perception of psychosocial implications of tubal sterilization. For some participants, sterilization was followed by peace of mind and body; however, others experienced a range of complications stemming from their body, psyche, surroundings, and their relationship with healthcare providers. Any practitioner conducting procedures in a similar setting should be aware of the above-described problems and consider such potential complications in the management plan of clients.

In the future, studies are needed to shed more light on the involvement of extended family in making the decision for tubal sterilization, the ethics of involving third parties in this decision, the place of patient-centered care in a rural context, and the determinants of post-tubal ligation outcomes in such settings. From a practice perspective, the time and methods devoted to preparation for tubal ligation need to be revisited in light of the current study. Moreover, appropriate involvement of other persons could be determined after full participation of the client in the decision-making process.

## List of abbreviations

QOL: quality of life.

## Competing interests

The authors declare that they have no competing interests.

## Authors' contributions

PML conceived and designed the study, collected data, analyzed and interpreted data. JH designed the study, interpreted data, and revised it critically for important intellectual content. LNL designed the study and revised it critically for important intellectual content. All authors read and approved the final manuscript.

## Authors' information

PML: is a family physician, currently volunteering under the United Nations Volunteer in Malawi and working as HIV Zonal Supervisor with the HIV-AIDS department of the Ministry of health. JH: Professor, Chief Family Physician specialist and head of the department of Family Medicine at the University of Pretoria, South Africa. LL: Surgeon and dean of the Medical school, Université Evangélique d'Afrique, Bukavu, Congo.
